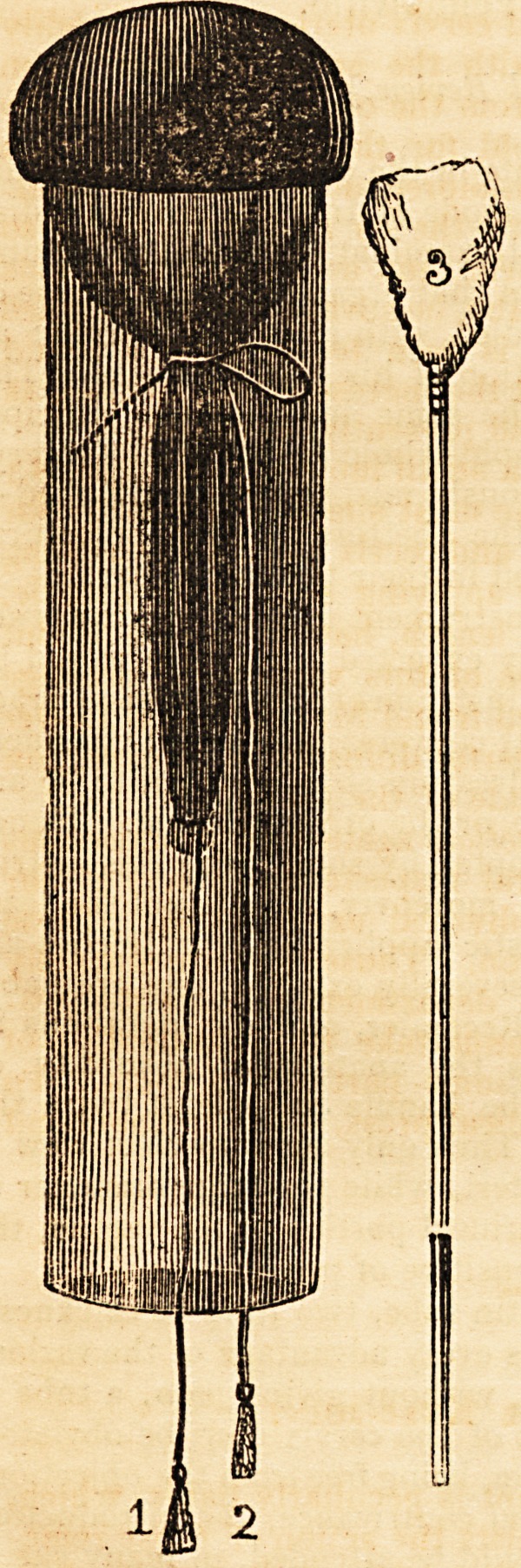# Extra-Limites

**Published:** 1839

**Authors:** 


### EXTRA-LIMITES.

Dr. Furnivall on the Ætiology and Treatment of Phthisis,—in answer to a Critique in the British and Foreign Medical Review.

Sir,

*Hertford, May* 22, 1839.

May I beg the favor of you to insert, in your Number for July next, the following observations on a Bibliographical Notice, which has lately appeared in the British and Foreign Medical Review, of my Practical Essays on the Treatment of Consumptive Disorders, &c. I should have addressed myself to the Senior Editor of that Review, had I not been warned by what has recently occurred to Mr. Lizars, from whose statement, it appears probable, that if I had appealed in the name of justice, for an insertion of my Strictures on the wretched scribbler's tissue of misrepresentation, I should have been asked for payment. To string together foul misrepresentations, or biting calumnies, in order that the traduced writers may be anxious to pay for the insertion of their vindication, may be one way of getting a little money—but it is not a very respectable one. Is it that this Editor, in default of a satisfactory circulation of his Quarterly Galimathias, has adopted for his motto, Rem, quocunque modo rem, and for his rule of conduct:—

Fear not to lie, 'twill seem a sharper hit,
Shrink not from calumny, 'twill pass for wit.

The only number of this Review that I have seen, or haply ever shall see, is No. 14; but, after your statistical calculation, and clear exposure (*see Medico-Chirurgical Review*, July 1838) of the wretched system of puffing, and of the title-page trickery—in promise, mighty, in performance, nothing—which has been pursued by this worthy and his colleague, one might have supposed that they would have been more cautious in throwing stones at other people—for these barking curs have by this time, it seems, had their ears well torn—but no, it is to be feared, that the same conceited vanity which has deluded them into setting themselves up as censors of others, will unhappily induce them to go on in their present miserable course of "cushioning, garbling, or positively misstating the opinions of writers" whom they may review, while they will also continue to "retail their own peculiar crotchets, or vent their *interested pique*, under the garb of impartial and conscientious censors of truth."

No man is more disposed to defer to sound and impartial criticism than myself—but an infamous system, such as this is, ought to be put down; and when periodical depredators like these men, under the disguise of critics, infest the highways of knowledge, to attack the peaceable traveller, and exult in any dismay which their assault might occasion, I for one will say—

While these are censors, 'twould be sin to spare;
While such are critics, why should I forbear?
Why not, in truth, the gauntlet cast at once,
To Scotch Marauder, or to Southern Dunce?

Reviewers are but individuals, and often very obscure ones too. These scribblers set themselves up to examine and denounce, without proof given, the claims of other writers—what will become of them when their own claims are examined? What right have they to seat themselves in the chair of criticism? Are they not self-called-for and self-appointed, or else, are they not the mere literary hacks of a bookseller's enterprize? Can they prove that such a publication as theirs was wanting? Can they prove that they effect any more good to our profession than your long-established Journal has always done? Can they prove that they effect any good at all, which is not much better done in your periodical? Does not the very number of books, which they profess every quarter to review, give evidence that those who take in their Review must be deceived?—for no men can effectively perform what they promise to do within the time and limits proposed by them. For myself, I should have pursued the even tenor of my way, neither roused into reply nor excited into recrimination, by my critic's one-sided attack—for two reasons—the one, that I am, at all times, disinclined to acrimonious and needless controversy (needless, because truth will always make its own way, in spite of all opposition)—the other is, that I feel I shall be serving the interests of my critic, by pointing attention to his little-read pages—still, at the risk of "throwing a cruel sunshine o'er the rogue," I have resolved not to allow his assertions to pass uncontradicted—lest, if I did so, I might be considered as acquiescing in their correctness. I have, in addition, the important object in view of pressing upon my professional fellow-labourers—upon practical men—the value of the system of treatment, taken as a whole, which I have adopted, and which, I believe, will lead to more successful results than have been hitherto observed.

That time, and the cautious experiments of others, may prove the soundness of my directions, I sincerely hope—in the meantime, I can only urge to the trial—and say, again and again, his utere mecum.

My critic commences his attack very insidiously, and not daring to make an open charge of improper motive, he confines himself to an insinuation as dirty as the source whence it sprung. He then charges me with want of novelty. Now, supposing this charge were true, has this Reviewer yet to be told, that time may be much better employed in learning how to use our old remedies than in seeking after new—in consolidating and improving what we already know, rather than in running a wild-goose chase after a philosopher's stone, in the shape of novelty?

Did I boast of novelty or originality? No. Had my Reviewer done me but justice, he would not have dishonorably suppressed that part of my Preface, in which I stated "that, although the results of practice which I was about to publish, had been worked out by myself, and were so far original, yet, as I had not lately had extensive opportunities of professional reading, I doubted not but that all I had to state, must probably enter into the aggregate of received or recorded opinions, as it may have sprung out of them." Oh! gentlemanly Reviewer!!

My Reviewer's next charge would seem to many, highest praise—namely, that my little practical work may be regarded and used as an abridgement of *two Treatises*—one by Dr. A. Combe on Hygiene, the other by Sir J. Clarke on Consumption—although it is said, that I have only slightly referred to the former, and not mentioned the latter at all. This latter charge, if it were true, would touch me sensibly; but, with respect to Dr. Combe's work, when I took one extract from it, and I took but one, I acknowledged the obligation specially—and is it not too bad that the Reviewer has again wilfully and dishonestly suppressed, indeed has not even alluded to these words at page 6 of my Preface—"The hygienic precepts have been succinctly compiled from the *numerous* writers, *British* and *Foreign*, who have published on Hygiene." He has, moreover, suppressed the special, very important, and little attended to, application of a certain part of hygienics to the pathological states, afterwards described by me. With respect to Sir J. Clarke's work, I beg to inform the Reviewer that I have never seen it, still less perused it—but I have seen it advertised as a Treatise on the Treatment and *Cure* of Tuberculous Diseases. I have no more knowledge of it than could be acquired in a perusal of a good analytical critique on it, inserted in one of your numbers. I fear my Reviewer's morality is of so low a nature, that he will hardly give credence to this assertion, because he may think it suits my interest to make the avowal—if so, gentlemen can have no argument with such a man—for him, honor, and truth (which indeed ought to be but another name for honor), and the courtesies of polished life, exist in vain. After a careful perusal, not to say study, of the works of Bayle, Laennec, Andral and Louis, I felt that the only way to arrive at sound practical results, was to study for myself, at the bedside of the patient, and not incur the danger of bias from any one.

My Reviewer then seems to talk in a patronizing Malvolio strain—that "but for one unfortunate pretension" (to which I shall allude anon) he—no—"we are not sure but we might have *quietly* recommended Dr. F.'s little book to the class of *general readers*, without any depreciating criticism"—having previously said that "the small volume contains much that is true and valuable, in regard to the preservation of health and the prophylaxis of disease, and will be useful," &c. The exquisite magnifico!! What shall I do, now that I am deprived of the light of this scribe's patronage? Really, Sir, this fellow's self-conceit were enough to move one's wildest laughter, if it had not been accompanied with such foul suppressions and misrepresentations. Has the British and Foreign Medical Review any general readers?—one should think it unlikely:—would then his praise have sold a single copy? "Self-conceit doth make a rare turkey-cock of him—how he jets under his self-advanced plumes!" As Pope says—

> Whatever Nature has in worth denied,
> She gives in large recruits of needful pride.

Sir, if my work really be so valuable as he describes it, it was his duty as a Reviewer to have recommended it. Perhaps he is determined to give no praise, except to his own friends.

> "Nul n'aura de l'esprit, hors nous et nos amis."

And now, Sir, to the Title-page. I assert, in the face of the Reviewer and, with the utmost deference, before the whole profession, that I believe the allegations therein to be perfectly well founded, and as well proven as the nature of the malady will allow them to be. But first, let me protest against the present general well-known tendency amongst medical men, to despair in the treatment of Tubercular Consumption—the word success, it seems, is to be tabooed, notwithstanding the revelations in morbid anatomy—what is, what must be the consequence? The consequence must be, a relaxation or cessation of those efforts to cure this disease, which are indispensable. The road to success has never lain through despondency or despair. Banish the word success from our mouths, and the thing itself, as applied to this malady, will be banished from our bosoms. What right have we to assume that a Beneficent Deity may not grant to persevering efforts, a sure knowledge of the means whereby a cure may be effected. Mais revenons à mon mouton. My Reviewer says, I have not given proof of "cure properly so called." Now, the truth-evading scribe is here quibbling upon words, and is moreover putting words into my mouth, which I disclaim. My pretensions are confined to successful treatment, as distinguished from "cure properly so called." I restrict myself to the support of my patient under the disease, to conducting him or her through the attack without loss of life, and, in the earlier stages, to the prevention of an attack that may have been threatened. I am more a man of things than words—res non verba quæso—but I see and feel that a difference exists between the words, successful treatment, and cure properly so called. At least under that impression I have written. I have described the cases in which I should entertain hopes of success, and I have not omitted to mark down, though hastily, a number of exceptions, which form but too long and melancholy a list of incurables. My Reviewer says he has read my book attentively—if so, he has read page 10 of the Preface, and elsewhere in the work—yet, not a word does he say thereupon. This is not fair.

The Reviewer, "fragili quærens illidere dentem," fixes on what he thinks the weakest point, and denies, *with sorrow*, that I have adduced satisfactory evidence of phthisis pulmonalis, in its early stages. Well does he know how easy it is to bring such a charge, and how difficult it often becomes, so to rebut it as not to leave a peg, whereon to hang a doubt—and here again this uncandid scribbler, this unjust judge, has suppressed these words at page 191: "In practice, all the evidence we can adduce, exclusive of the post-mortem appearances, is, and must be, of a negative character—for all we can say is, that we have had patients suffering with all the usual signs of tubercular deposit, who have recovered their health without going through the stages of softening, &c." The diagnosis which I have given of phthisis at this stage is corroborated by Mons. Louis, and every other practitioner of experience; and my professional brethren will agree with me, that when a person comes to the medical man labouring under the symptoms described at page 148 of my work; when there is further corroboration from the previous history; and there is an hereditary tendency—we shall hazard little, if we make up our minds that a case of tubercular consumption is before us, and we shall *do a great deal* if we can relieve our patient from his threatening symptoms. The pictures which I have drawn of symptoms have not beeen extracted from books; but have been taken at the bedside and prescribing table.

The Reviewer says, that when evidence has been given in my work, both of the existence of phthisis, and of successful results—which latter, he chooses to term "so-called cure"—this special pleader evades the difficulty by saying that the cure is recent, &c. Finally, he slurs over my treatment—says it is common: now so far is it from being common, that although the medicines prescribed are all long known, I believe it is, when taken as a whole, and applied to the pathological states described, very different from the routine, and in several respects, quite peculiar. The principles of the treatment are not common—my use of mercurials is not common, for they are usually strictly prohibited in phthisis—my mode of sparing the strength and of checking the colliquative perspirations is not common—my use of soda is not common—my reliance on ipecacuan in some cases of hæmoptysis is not common—the way in which I treat amenorrhœa in phthisical habits is not common—and my suggestion as to the treatment of *obstinate chlorosis by emetics of muriate of soda, with my reasons at page 221*, together with my treatment of opacities of the cornea, and of strumous conjunctivitis, by the external use of iodine, are, I submit, novelties highly worthy of trial. But, Sir, novelties or no novelties, I have no hesitation, in the full strength of an honest conviction, to repeat, that I believe my title-page contains no assertion which has not been as fully borne out as possible by facts, and by facts too published in my book.

Permit me to conclude with a few remarks on the probable proximate morbid condition in the phthisical diathesis. It is of no practical use to give this condition a name, and call it tuberculous cachexia—we want to know its nature if we can. Theory is notoriously dangerous ground to tread upon, and few persons can be more jealously watchful against the bewildering yet fascinating encroachments of hypothesis upon the human mind than myself—still there is no wisdom in continuing the old mill-horse round in treating this malady; if any success is to be expected, it can only be found by varying the present nearly uniformly fatal system of therapeutics in this disease. In my Essay I have pointed to *facts* alone—for the *practice* there recommended is *quite independent, yet corroborative* of the proposed theory.—See pages 172 et sequent.

It long ago occurred to me, that the proximate morbid condition in the phthisical diathesis is probably a fulness or congestion of the great venous sinuses—especially of the pulmonic—and that this state would seem to be caused by defective energy of the organic system of nerves; whence would result an imperfect performance of the functions of the capillaries, cutaneous, pulmonic, and hepatic. We find accordingly the indicia and proofs of these states, in the obstinate coldness of the extremities, the chilliness and almost exsanguine state of the skin, in the frequently attending costiveness, and in the cough and dyspnœa, with its accompanying depression at the præcordia and sternum. This state forms the condition of persons who are in delicate health, or who are threatened with consumption. I have denominated it the venous congestion of the premonitory stage; nor is this merely a name—for it points to practice. This state may be accompanied with many alarming symptoms—a quick pulse—dyspnœa—a dry cough—and even *loss of flesh;* for the final assimilation of the food is checked, if not stopped. This loss of flesh is sometimes one of the earliest symptoms, and may exist without any marked sign of thoracic disease. Under these circumstances, when exercise to a salutary extent cannot be taken, when healthy respiration cannot be performed, and when the great carbon removing auxiliaries, the liver and skin, do not act as they should do, one must not expect an efficient depuration of the blood—carbon will accumulate—and more particularly it will accumulate where, by position, the lungs are least moveable, viz. in the upper lobes. Thus both a pabulum and a nidus for tubercular matter are afforded in this carbonized blood, which by its sluggishness of current is rendered additionally fit for morbid deposition. This tendency to venous congestion in phthisical habits, when it exists predominantly in the abdominal circulation, and portal system of veins, may give us a glimmering in explanation of the hitherto inexplicable connexion between fistula in ano and phthisis; and may shew how it is, that if we cure this fistula, without establishing after cure some system of prevention of disease, consumption often follows—a fact well known to practitioners. With respect to the existence of too much carbon in such blood, the only experiments which bear upon the subject, as far as I know, are those of Dr. R. Clanny—a physician practised in analytical animal chemistry. He submitted to analysis—20 ounces of blood, taken in vacuo from the arm of a young man labouring under phthisis—14 ounces contained one cubic inch of *free* carbonic acid gas; and from an analysis of 1000 parts, the following were the results:—water 787 parts; albumen coagulated at 160° Fahr. 95; colouring matter, 61; *free* carbon, 33; fibrin pressed and dried in the atmosphere, 19; *salts* and animal extractive, 5. Dr. C. made another analysis of the blood taken from a young woman highly phthisical, and there were found 31 parts of *free carbon,* with only 4 of *salts* and animal extractive in the 1000 parts.

Now if we compare this with healthy blood, we shall find an *excess of carbon* and a *deficiency* of saline matter in the blood of phthisical persons. The tendency of the former is directly sedative, while that of the latter co-operates sedatively by its deficiency of healthy stimulus. Accordingly we find debility, or (if I may so express myself) a lower grade of vital power, to be the constant characteristics or concomitants of the phthisical constitution. In nearly all the histories of tubercular deposition, we shall find that debility has been, in some way, previously induced, by exhausting discharges or diseases, scanty food, bad habits of life, &c.; while we shall also find, in many of the fatal cases, that death has ensued rather from the failure of the vital powers, than from the great extension of the disease, and its encroachment over the lungs causing asphyxia. How does a surplus of carbon affect the composition (chemical) of tubercular matter? It appears by the latest analysis, that this matter consists chiefly of albumen, with various proportions of fibrine and gelatine. Now albumen contains nearly 53 per cent. of carbon. Do not these circumstances form a tolerably strong chain of presumptive evidence to the effect, that the probable morbid alteration of the blood in phthisical persons consists in an undue predominance of carbon in it, and of carbon in a free state too? while at the same time there exists a deficiency of the usual saline stimulus in it.

The prophylactic, if not curative indications, become evident, to equalize the circulation between the interior and the periphery—to restore the functions of the skin—to elicit an increase and more general diffusion of the animal temperature—to regulate the hepatic and other secretions—to try to alter the morbid condition of the blood. These are the objects to be *aimed at*, although we may not always hope for complete success.

It is in this stage that the system followed by many, of ordering low diet and of prescribing bloodletting and digitalis for the quick pulse, &c., is very pernicious; and almost sure, by the induction or increase of the debility, already existing, to add to the very evil we are endeavouring to remove. Judicious hygienic measures, with especial attention to the skin—mercurials, judiciously and cautiously administered—gentle tonics—counter-irritation—and *sodaic salts*, become our most useful means of treatment.

But, excluding cases of general tubercular infiltration, let us take a more advanced case, wherein tubercles have been already deposited—and further, let us suppose that they, like other unorganized bodies, have from the application of some exciting cause, begun to cause an irritation in the circumjacent pulmonary parenchyma, another kind of congestion then arises—the active sanguineous congestion of Andral and other pathologists. We then have a specific disease with its certain defined limits, and a general affection of an inflammatory nature. We now know, that if the tubercles have softened to any considerable extent, a way must be made for their exit from the affected spot; at the same time, we must take care to prevent the increase of the specific disease by an extension of the tuberculous matter around, in, and on the suppurating surface. Hence it is of primary importance to resolve this active congestion as soon as possible, and with the *least possible cost* to the vital powers; for I have little doubt, that many of the instances of rapid disorganization of the lung which are met with, originated in this way; namely, by an extension of the specific disease over the common suppurating surface, which the active congestion above alluded to had produced.

When this active congestion has been resolved, or when suppuration has freely set in, and by its continuance for a certain time has removed the inflammatory state, then our plan must be altered immediately, and a tonic plan instituted, though with the utmost caution and watchfulness. The practitioner must be always on his guard against intercurrent pneumonia or inflammatory complications which may occur at any time—and he will find, that some cases will require the lancet repeatedly, others not at all. In my hands, mercurials have enabled me often to dispense with bloodletting—while my mode of checking and stopping the exhausting perspirations will be found very useful. The difference between this mode of treatment and the present fatal one, of repeated small venesections, &c. is very great; and, with the utmost deference, do I propose its adoption by the profession in preference to the ordinary vague, uncertain, and empirical system.

Mais encore une fois à mon mouton. I will now leave my Reviewer to weave his quarterly woof of tinsel; and will conclude with telling him, that he will be very ill-advised if he do not decidedly change his mode of reviewing—that when he feels obliged to condemn, he had better always find room for a fair exposé of his reasons and evidence—that although he may be a parasite-writer himself, or a mere redacteur from the French, other persons may be practical writers—that the venom of the dart is contemptible without some little vigour in the bow—and that the morals of criticism are included in candour, modesty, and good breeding.

I am, Sir, your's, &c.

J. J. FURNIVALL, M.D.

### Dr. Osborn on Dropsies.

Mr. Editor.—Since the publication of the second edition of my small Treatise on Dropsies, I have refrained from animadverting on any of the manifold observations, remarks, reviews and lectures which have appeared on the subject of coagulable urine, being convinced, much as I differed from several of them, that truth would at the end prevail. In this conviction I am strengthened by the late publications of Drs. Christison and Rayer, who, although proceeding on different paths, yet tend to unite at the point to which I contended the original proposition of Dr. Bright would ultimately be extended, namely, that albuminous urine is produced by, and is therefore a symptom of, diseased kidney.

It were well if those who use the word functional as opposed to structural would previously give a definition, and state if they mean by it anything more than temporary or of short duration as opposed to permanent, and if so, what it is. Is ptyalism a functional affection of the salivary glands because it is produced by apparently insignificant means and may last only for a short time, in any other sense than that of short duration? and can we conceive a gland to secrete in any unusual manner without some alteration in the state of the secreting organ which, however transient it may be, is yet structural as long as it lasts? To assert that ptyalism is a functional state of the gland, is merely to assert that it is a transient affection; but if from this fact, that ptyalism is sometimes only transient, we were, by availing ourselves of the ambiguity of the word functional, to jump to the conclusion that ptyalism is not an affection of the salivary gland at all, we should prevail with but few to agree with us, and yet a course exactly similar is attempted, by those who have endeavoured to disprove the connection between granulated disease of the kidney and albuminous urine.

There is no doubt that some have described kidneys as healthy although in the first stage of granulation. The only mode of avoiding this error is, by appealing to my test of injecting the kidneys. As the granulated structure is impervious to injection, this simple experiment ought always to be appealed to before a kidney which has secreted albuminous urine is pronounced healthy. In proportion as the body of facts to prove the connection is great and indeed overwhelming, so ought the scrutiny to be cautious and severe, before an instance is admitted to the contrary. Instead of pursuing the investigation according to this obvious law of induction, some have shewed a singular avidity in collecting opposite facts which, even if well proved, can *now* be only considered in the light of exceptions to a generally true proposition. Those gentlemen have displayed (not scepticism, but rather) an eager credulity for such facts, in proportion to their rarity and consequent improbability. For them, the slightest *on dit* has been quite sufficient, as I could shew if I entered into details, while the strongest evidence, and especially that afforded by injection of the kidney, is necessary to prove what they require. The individual who asserted that he found a fly in the centre of a marrow-bone was justly called on for proofs more decisive than would be required in an ordinary occurrence, and, after all his efforts, there must have been doubts and misgivings on the minds of many, as to whether the surprising fact might not have arisen from *some mistake*.

I have all along admitted that the albuminous secretion, in some instances, occurs in a transient form, but, as such have not been compared with appearances of the kidney itself, they prove nothing as to the granular deposit, and only shew that the disease is not necessarily permanent, which I never meant to deny. The proposition which I think has been established is, that the granulated kidney always secretes albuminous urine, and that, when albuminous urine continues to be secreted up to the death of the individual, the granular structure is always found in the kidney. Let the exceptions to these propositions be brought forward to the statical and numerical standard, and see if, in the whole range of pathology, there be any one symptom of an internal disease to which a higher degree of credit ought to be affixed.

My object however, in writing these lines, is not to express my continued adherence to the proposition now mentioned, but to clear myself from an error which Dr. Christison, in his late work, has attributed to me, and which, if suffered to remain uncorrected, would cause me to appear as one of those braggadocios, too common, alas! in our profession, who suit their statements to their own wishes or the prevailing tastes of the public. *Populo ut placerent quas fecere fabulas.* He says that I have alleged, in regard to dropsy, that I never failed to remove it, whenever the entire surface was restored to a perspiring state. Now Dr. Christison has herein (no doubt through inadvertence) omitted to state, that *I limited the above proposition to cases in which there was no complication with other diseases.* Now such cases are unfortunately by far the most numerous; the patients, in the great majority (compare Dr. Bright's, Dr. Christison's, and Dr. Gregory's cases in connexion with mine), ultimately sinking under comatose or convulsive affections of the brain, gastro-enteritis, bronchitis, or chronic inflammation of the peritonæum. *But when not thus complicated* (those are my words, and to which I adhere), the cases always terminated by disappearance of the dropsical swellings if general perspiration was established.

It is a very agreeable amusement to witness the contortions of authors suffering under the inflictions of their reviewers, and, next to the pleasure of reading a severe critique, is undoubtedly that of knowing how the unfortunate author *takes it.* An English clergyman of considerable celebrity has suggested the plan of a periodical, to consist of reviews of works by the authors themselves, reviews of the reviewers, and lastly the reclamations of enraged or disappointed authors. Although I have no great cause of complaint with regard to my little book, yet I beg to suggest, with great respect, that (a certain proportion of) the pages of this Journal would be well employed in affording authors an opportunity of explanation or of defence, when unjustly attacked. The necessity of free discussion and of freely balancing conflicting statements, is surely paramount in medical science, which depends so exclusively on facts. If I were allowed to review myself in my second edition, I should have brought forward many topics which appear to me of far greater practical importance than that now adverted to. I fear that I have already trespassed too far on the patience of the reader, but not wishing to be handed down in the durable pages of Dr. Christison and of this Journal as a medical Thraso, and the author of such an unwarrantable statement as that every case of dropsy in which perspiration occurred was followed by cure, I shall feel obliged by being allowed the opportunity of contradicting it, and am, Sir,

Your very obedient Servant,

JONATHAN OSBORNE.

26, Harcourt Street, Dublin,

27th April, 1839.

---

### MR. BARRON AND MR. PETTIGREW.

*Pittville, Cheltenham, 26th April, 1839.*

SIR,—I have been induced, at the suggestion of a medical friend, to transmit to you the annexed Copy of a Correspondence which I have recently had with Mr. T. J. Pettigrew, author of The Medical Portrait Gallery, and if, after perusal, you see no objections, I shall consider myself much obliged by the publication of the following Notice in some conspicuous part of the next Number of your Journal. I have the honor to be, Sir,

Your most obedient Servant,

THOS. BARRON.

Dr. James Johnson.

"*Note.*—A Correspondence betwixt the Representative of the late Mr. William Barron and Mr. T. J. Pettigrew, has been sent to the Editor, relative to an acknowledged error in the spelling of his father's name, in a Memoir of Dr. Baron in the first volume of 'The Medical Portrait Gallery;' and as Mr. Pettigrew has refused to correct the mistake, by the publication of an explanatory note, on the ground of its being a deviation from the established order of his book, the Editor is authorized to state that the alteration in the spelling of Mr. Barron's name was not authorized in the Memoir above-mentioned."

☞ As the Correspondence is too voluminous, and cannot be interesting to any but the parties themselves, we have limited ourselves to the "*Note,*" which contains the pith of the whole.—Editors.

---

### On the Advantages of a Speculum Cushion and Cylinder. By *J. L. Fenner*, Esq. M.R.C.S. Pentonville.

[*For the Medico-Chirurgical Review.*]

One of the most important modern improvements in the art of healing, in this country, is the departure from the conjectural mode of treating obstinate diseases of the uterus, and the adoption of physical examination by the speculum, so as to bring the various changes which occur in that organ within the scope of ocular examination, and enable them to be studied with as much accuracy as the ophthalmic surgeon displays in the investigation of his peculiar cases. The advantages of the speculum uteri arise from the same indisputable principle which has ever been acted on in the practice of ophthalmic and general surgery. By its employment cases which had been previously mistaken or overlooked—sufficient in number and importance to silence the abettors of the conjectural system of treatment—are daily becoming revealed in their true character.

On the introduction of a comparatively new instrument into the *private practice* of medical men, it might have been naturally expected that there would be a variety of opinions relative to the form of the instrument, and that each practitioner would extol that with which he was most conversant, without considering the respective merits of each, or what desideratum still remained to be accomplished by the inventive faculty in either one or the other of them. Hence Ricord's two-bladed speculum is the *only one* adopted by some; but this instrument is made but of *one size* for *every case,* and, moreover, affords not the least protection to the surrounding parts when caustic applications or leeches are used. The three and four-bladed specula are severally extolled, and are liable to the same objections. The metallic tube of Recamier, and its imitations in glass, find their advocates also; these possess the anatomical absurdity of gradually increasing in circumference towards the handle to at least twice the size of the end in contact with the os uteri. Thus only an imperfect view is afforded of a very small portion of the cervix uteri, while the large diameter of the speculum is preposterously applied to the perineal portion of the vagina, the part the least disposed to dilate, and the most sensitive of pressure.

A tube of glass, or German silver, or of cast-tin tube, two lines in thickness, if made of *the same diameter throughout,* possesses every advantage of the various bladed specula; and if the difficulty of passing, without giving pain, a tube of sufficiently large size to expose the whole extent of the cervix uteri be obviated, it must be superior to any other form of speculum, being free from the objections already stated. Without the addition of artificial contrivance, the introduction of such a tube of sufficiently large diameter would be attended, in many cases, with pain, probably with injury. For the purpose of using a tube of requisite size with facility, and without pain, I attach an air-cushion in such manner that its soft elastic projection might previously produce dilatation, and, by overlapping, might protect the parts from the pressure of the edges of the tube, as seen in the accompanying sketch.

Small bladders, or the crops of poultry, partially distended with air, and disguised by being stained with orchil, answer the purpose of the cushions, and can readily be procured. The cushion is formed by twisting the depending portion of the bladder so as to force the air into its superior part, and then tying it with a silken cord in a slip-knot, leaving the end long enough to extend below the bottom of the tube, (*fig.* 2,) as represented in the sketch, ready for introduction, it is to be smeared with some unctuous substance, and then, being held in the right hand, the cushion is to be applied to the vulva with firm but gentle semi-rotatory pressure, and very gradually passed on in the direction in which the cervix uteri has been previously discovered, by the taxis, to be situated. As soon as the speculum, with its cushion, has passed the perineal portion of the vagina and has entered the pelvic cavity, the cord (*fig.* 2.) is drawn to untie the slip-knot, and enable the air which distended the upper portion, and formed the cushion, to rush into the depending part; when, by means of the cord (*fig.* 1) the tube is left clear, by the bladder being withdrawn, disclosing a circumference of the os and cervix uteri proportionate to that of the tube.

1 2

The speculum cushion was invented for the especial relief of cases of irritation and inflammation, which thus may be examined without producing pain, and a consequent increase of symptoms. In cases where little irritability exists, I adopt a more simple invention, but upon the former principle of producing previous dilatation before the edges of the tube press on the vaginal structure.—A piece of box-wood of two inches in length is, by a turner, accurately fitted to the inner edge of the tube. One half-inch of the end is conically sloped to a rounded extremity, which projects during the introduction of the instrument, to dilate the vaginal structure before it comes into contact with the edges of the tube. The thickness of the edge should never be greater than two lines; and, if rounded, its obstacle is scarcely perceptible to the patient. By boring a hole in the other end of each cylinder, a common handle may be used for various sizes, which may be removed when not in use. I trust that this will be rendered sufficiently intelligible by looking at the former sketch. The part overlapping the edges of the tube is supposed to be removed, and the cushion to assume a more conical form. Where the slip-knot appears may be supposed to be the part of the cylinder where the hole is made to receive the common handle; and, parallel to this, if the stem (*fig.* 3) be the handle, the cylinder may be removed as soon as the tube has passed the perineal portion of the vagina.

My speculum case contains tubes of *three sizes two lines in thickness, and five inches in length.* The diameter of the smallest size which I employ is one inch, including the thickness of the tube; the second is one inch and a quarter; the third measures one inch and a half in diameter. Besides the advantages of introducing by this mode the tubular speculum, with facility and without pain, and gaining by it every possible information, it possesses peculiar intrinsic merits such as the following:—The whole extent of the mucous coat of the vagina can be explored most accurately, and passed in review under the eye as it is pressed into the cavity of the tube. I lately attended a case of leucorrhœa with one of the most able advocates of Ricord, and discovered, by means of the tube, three chaps on the posterior part of the vagina, which could never have been seen with a bladed speculum. The minor operations so frequently required in the treatment of diseases of the uterus, are also rendered more efficacious and safe by the use of the tube, which may remain a sufficient time without causing any pain. The application of nitrate of silver, ten grains, in a drachm of water—most useful in abrasion or ulceration of the os and cervix uteri—may be readily used. The injecting of the cavity of the uterus with the same solution, when the peculiar glairy discharge is seen to issue from the organ, may be readily accomplished, not by the instruments usually sold for the purpose, but by a silver canula fitted to a small bone syringe: the unimpregnated uterus will contain but about half a drachm of fluid. Leeches to the os and cervix uteri, so valuable in congestive and inflammatory engorgement, and at the critical periods of amenorrhœa and dysmenorrhœa, may, by the tube, be advantageously applied to a large circumference; but where the os uteri is seen to be open, I would suggest the application of a piece of lint to prevent the ingress of the leeches, as troublesome though not dangerous symptoms would presently supervene.

In the above engraving I have given a sketch of a small mop or brush (*fig.* 3,) one of the most useful and convenient, though the most simple instrument for removing mucus and other substances from the os and cervix uteri, and enabling its structure to be clearly examined; and also for applying solution of caustic, &c. It is a skewer of wood, about six inches in length, having a piece of lint or wadding tied round the top. For the suggestion of this valuable appendage to the speculum case I am indebted to my talented friend Mr. Jones, of Lower Brook Street, whose indefatigable labours and cheerful diffusion of information on this neglected subject, entitle him to the gratitude of the profession.

It must not be concealed that there are *moral objections* against the promiscuous use of the speculum uteri strongly felt by several respected members of our profession who, consequently, are opposed to physical examination. These objections are entitled to the utmost consideration. Those who are sincerely anxious to promote the objects of science in this department will see the propriety of adopting an established rule—never to undertake the examination of a patient unless a female friend be present, or in some part of the room. To public teachers and the talented editors of the medical press, I beg to commend the advocacy of the moral view of this subject.

---

### Mr. Meryon on Disease of the Knee-joint.

There is a morbid condition to which the knee-joint is peculiarly liable, which, inasmuch as it is usually of long duration, and resists the action of those remedial agents which are usually employed, is often the cause, rather of vexation than of anxiety; but in several cases which I have witnessed, the accompanying pain has been so severe as to excite some degree of apprehension both in the patient and surgeon. These latter instances I have noticed more especially in young females, who, from various exciting causes (an erroneous system of physical education not the most infrequent) have suffered greatly from constitutional irritation, which has eventually appeared to concentrate itself in the knee-joint, and to be productive of the various characteristics of acute inflammation: viz.—pain, redness, heat and swelling.

The character of the pain is generally described by the patient as being a deep-seated, aching, and sometimes grating, as though the articulating surfaces of the joint were rough. This latter sensation is of course felt only when the extremity is moved. If there be superficial pain it is generally referred to that part which is situated between the ligamentum patellæ and the course of the sartorius muscle. The redness which I have noticed may have been produced in many cases by previously employed counter-irritants, but in most instances there has been a diffused blush over the whole surface of the joint, manifestly dependent on a congested condition of the capillary vessels. The sensation of heat is mostly referred to the deep-seated parts, and the swelling is unequivocally attributable to an accumulation of fluid in the synovial membrane of the joint. Sometimes I have imagined that the small bursa behind the lig. patella has been distended with fluid from a feeling of fluctuation and apparent puffiness on either side of the ligament; but in most instances the fluctuation is too widely diffused to be owing to that cause simply.

The general health may or may not be much affected; but constipation of the bowels succeeds as a matter of course from want of exercise.

Mechanical injuries done to the knee are exceedingly apt to leave all the foregoing symptoms, and in very many cases they are not in the least degree influenced by the usual curative agents which are generally employed. The immediate effects of such mechanical injuries are doubtless most philosophically and efficiently treated by leeches, &c.; but as all the symptoms frequently continue in spite of the frequent repetition of such remedies, together with long confinement and absolute rest, it becomes a matter of no small importance to have a remedy which shall effectually subdue them.

Such remedy may, I apprehend, be confidently looked for in galvanism, applied as I will hereafter describe; but being still engaged in collecting materials for the determination of those morbid conditions which may be relieved by the application of the remedy in question, I refrain at present from discussing its modus operandi, trusting to some future opportunity of elucidating this point when I shall have determined more satisfactorily the exact amount of influence it may have in producing the various results which I am about to describe. My object now is to make known the *invariable success* which I have had in such cases with the hope that the means whereby such result has been produced may be made more generally useful.

Miss B. æt. 17, of Bourn in Lincolnshire, had in the Autumn of 1836, an attack of inflammation of the knee-joint, unprovoked by any previous injury. For this affection, leeches and blisters had been repeatedly applied during the Winter, and medicines had been given with a view to improve the general health; but with little or no benefit. In the Summer of 1837, she was sent to the sea-coast to try the effect of sea bathing, and returned without the least amendment. In Nov. 1837, she was sent to town and placed under my care. At this time the knee was so much swollen as to present a globular appearance, and on each side of the ligamentum patella a distinct fluctuation was to be felt extending over a considerable surface on either side—the pain was deep-seated and very acute, so much so as frequently to prevent sleep. The bowels naturally costive had become obstinately so; requiring the most drastic purgatives to relieve them, and the general health was much impaired in consequence of long confinement.

Previous to the outbreak of any disease in the knee, and for some time after, there had been a considerable degree of bodily derangement which was benefitted by medicinal agents; but with no accompanying amendment in the condition of the knee. On her arrival in London, I again attempted, in concert with Sir B. Brodie, the improvement of her general health, until the 17th of January, 1838, when, from previous knowledge of the effects of galvanism, we agreed that it should be tried. From this time forth until the 20th of the following month I continued to galvanize her every alternate day—and at the expiration of that time the knee was perfectly well. The pain began to diminish after the first application. After the third or fourth, the bowels were relieved daily without the use of aperients, the swelling gradually subsided—as did also the feeling of heat; and at the time the remedy was discontinued the general health was very greatly improved, notwithstanding that little or no exercise had been taken in consequence of the very cold weather. Before the end of March, however, she had been to three or four balls, at which she had danced without the least difficulty, and from that time to the present the knee has been perfectly well, and the general health very good.

The foregoing was manifestly a case which every surgeon would unhesitatingly attribute to an erroneous system of physical education: the following appeared to be more dependent on hereditary tendency, inasmuch as it was also unprovoked by any violence, and several members of the family have suffered at various times from diseases of the joints.

Miss L——, æt. 30, was affected during the greater part of 1837 with a disease of the knee, accompanied with much deep-seated pain, a sensation of heat, and some inconsiderable swelling. In this case also the bowels had become torpid, the appetite diminished, and the general health much impaired. For these symptoms she had been treated medicinally for many months, and during the greater part of the Summer had been at Brighton and other places on the S.E. coast, but without the least improvement. On the 20th of November, 1837, I galvanized the knee, and continued to do it every alternate day until the 28th of December, when it was perfectly well, and appeared to be as strong as the other—the swelling had gradually subsided, and the bowels, as in the first case, had become regular. Still the general health was not so greatly improved, and continued more or less affected, till at last, in March 1838, the other knee became affected in the same way that the first had been; but not so severely—either as regarded the pain or swelling. On the 22d of April, I recommended galvanism, and continued it every alternate day until the 22d of May, during which time the general health had been greatly improving; and when the remedy was discontinued, she was perfectly well—the knee in a sound and healthy condition—nor has there been the least return of the ailment since.

That diseases of this description may arise without any apparent cause, and be relieved by the same means, may be inferred from the following case.

—— Everington, Esq. had been complaining for six weeks or more of much deep-seated aching pain, unattended with swelling, but producing some degree of difficulty in walking. The case was just such as would induce almost every surgeon to apply leeches, and twelve were in fact directed to be applied by Mr. Liston; but the pain continued unabated. Feeling confidence in my remedy from frequent observation, I recommended the trial of galvanism, which was accordingly done. The second application produced very considerable relief. The third, I have every reason to hope, completely cured it, for I have heard nothing more of my patient.

The same symptoms which I have described as arising in the three former cases spontaneously, are often the result of injury sustained in or about the joint, from falls, blows, and other mechanical violence.

T. Laurence, Esq. æt. 32, had, in 1829, been thrown from his horse and in consequence of falling on one knee had suffered much pain in the joint, with considerable swelling, heat and redness. Leeches were at first applied, but with no benefit; they were frequently repeated, as were also blisters, fomentations, lotions, embrocations, baths of every description, still no relief was afforded to the disabled knee. In this way he continued upwards of three years, occasionally walking a little, but with difficulty, and occasionally completely laid up. From long confinement his bowels had become constipated, and from the constant use of aperient medicines the stomach and duodenum were so much affected that considerable pain and flatulent distention were occasioned whenever he took his food. On the 18th of Jan. 1833, I recommended the use of galvanism, to which he readily consented, and every alternate day I made the application until the 9th of May following, when the knee was perfectly well. In February he was enabled to walk from his house in the New Kent Road into the City, which he had not done for three years before. Notwithstanding the local improvement in the knee the affection of the stomach and bowel continued for more than a year after, occasionally relieved as the knee showed a little disposition to irritation. In April, 1834, the pain and swelling of the knee recurred almost as violently as ever; the same remedy was applied daily until the 24th of May, when it was discontinued, the knee being quite well. Since that time there has not been the least disposition to any morbid action about the part. There is, I understand, from time to time a feeling of weakness in that joint, but no pain, and generally he can walk and ride as well as ever he did, and without feeling more fatigue or inconvenience in that knee than in the other.

T. Pix, Esq. of Peasmarsh, in Sussex, had a fall when shooting, early in 1833, and hurt one knee, from which time until he applied to me, there had been considerable swelling, some degree of heat, a constant, deep-seated, distressing, grinding pain, as though the articulating surfaces of the femur and tibia were rough. On the 20th of April, 1834, I first applied galvanism, and on the 20th of May the knee was perfectly strong and well, and still continues so. In this case leeches and blisters had been repeatedly applied, but with little or no benefit. Since being so galvanised I hear that he walks as well as ever when out shooting, and runs in the cricket-field with as much facility and as little fear as though the knee had never been affected.

— Thomson, Esq. of Chelsea, applied to me in Oct. 1838, in consequence of an injury which he had sustained in the left knee on the Easter Monday previous, whilst playing at trap-ball; the ball having struck him. On examining the part I found considerable swelling of the joint, some degree of heat and redness, and he described the pain as being deep-seated and of a grinding character. On the 10th of October I first applied the galvanic wires, and on the 26th of Nov. the knee was as strong and well as that of the opposite side. The remedy having been applied with occasional intervals of four or five days.

Job Heath, Esq. Borough, about four years ago, when attempting to make a sudden spring with a person on his back, felt a pain in one knee, and since that time he has experienced more or less pain but not much swelling. He had at first 28 leeches applied, and after them, two blisters; still the joint continued painful and weak, and has remained so up to the present time. I have now galvanised the part four times, and the pain has entirely subsided.

Such are the various conditions which I have witnessed from blows, &c. received on the knee, and in no instance have I been unsuccessful in relieving them. There is yet another case of the many which I have noted, and which I am anxious to describe from the peculiarity of its origin.

Mr. P. clerk of J. P., Esq. of Bermondsey, after a long-continued course of mercury, during which he had imprudently exposed himself to wet and cold, became affected with a very painful disease of both knees, accompanied with considerable swelling and complete derangement of the general health. Such had been his condition for some years, and during some few months before seeing me he had been completely confined. On the 7th of October, 1836, with the concurrence of Mr. Travers, I first commenced to treat the knees with galvanism, and on the 12th of December he was well enough to walk up to my house; since which I have lost sight of him, but have every reason to believe that he is now quite well.

In the whole of the foregoing cases, except the last, wherein I gave sarsaparilla, I carefully avoided all adjuvants in the shape of medicine, that I might be the more satisfied of the effect of my remedy.

There are some few phenomena which present themselves during the application of galvanism which tend to show its influence over the secretions, and prove that its action is by no means entirely local.

During the time of its application a copious perspiration *generally* breaks out over the whole surface of the body, the action of the bowels is increased when the remedy is used as often as on alternate days, and notwithstanding the apparent excitement, the pulse is not increased either in frequency or strength.

The perspiration appears to be perfectly independent of the pain produced, for in the third case which I have related, as in many others wherein perspiration is excited with great difficulty, it has appeared most copiously under the influence of galvanism and where pain has not been so greatly complained of.

The influence of galvanism on the action of the bowels has been more or less apparent in every case which has come under my observation; but was more particularly illustrated in Miss B. who, previous to being acted on by galvanism, took two drops of croton oil with ext. coloc. almost every night. The same effect was made manifest in the case of a nobleman whom I galvanised from May to July, in 1838, for swollen ankles resulting from gout. There was in this case some considerable deposition of solid matter interfering with the action of the joints, and therefore the swelling was not much reduced. There was, however, very great improvement produced in the power of locomotion. But what struck me most was the perfectly regular action of the bowels during the whole time of the application of the remedy in question, and for at least two or three months after, although for years previously not a week had been passed without aperient medicine having been required. Once during the application there was a threatening of gout (stiffness and puffiness of the fingers of one hand) after some little imprudence in diet, but, contrary to the usual custom, all the symptoms vanished after the next application of galvanism; whether or no in consequence of such application I will not venture to say; but certainly, inasmuch as it produces no vascular excitement, I should not fear the action of such remedy during the inflammatory condition of gout.

Supposing that these phenomena might be dependent on the direct influence of the remedy on the nervous system, I have in two cases applied it in neuralgic affections. In the first, there was partial paralysis of one inferior extremity with neuralgic pains in the very muscles which were so paralysed, which appeared to be those only to which the anterior crural nerve is distributed. In this patient not the least benefit was experienced; on the contrary, the pain was rather increased.

In the second case, the subject of which was a young lady who had for many years suffered from tic doloureux in the superior maxillary nerve, the pain was manifestly increased during the application of galvanism—and recurred as frequently as though no remedial agent had been employed.

### MODE OF APPLICATION.

The instrument which I usually employ is a simple galvanic trough, containing 50 pairs of zinc and copper-plates—each pair being about two inches square. To excite galvanic action, I have used two fluid ounces of concentrated sulphuric acid diluted with two quarts of water. The surface to which the wires are applied I keep moistened with a solution of common salt (muriate of soda) in water. The application I generally make every alternate day, and for about ten minutes each time—not by allowing it to pass through in a constant stream, but by quickly applying and withdrawing the wires. During the first few days there will be a degree of stiffness experienced, but this very quickly subsides; and in no case have I continued the application (except in the two last instances which I have related) more than a fortnight, without very manifest benefit.

EDWARD MERYON.

4, Bolton Street, Piccadilly.

---

### TO THE EDITORS OF THE MEDICO-CHIRURGICAL REVIEW.

*Aberdeen*, 17*th January*, 1839.

Gentlemen,

I have just read in your Review of the Transactions of the Medical and Physical Society of Bombay two cases of dysentery, in which portions of intestine were voided per anum. The second case, by Dr. Inglis, is said to be more curious than the first, since recovery took place. It is reported as follows:—" J. T. ætat. 40, admitted into the European Hospital April 22d, 1835, stated that for six or seven days he had been suffering from bowel complaint, with frequent and often ineffectual calls to evacuate the bowels. There was tenderness across the abdomen on pressure, with a slight heat of skin, and frequency of pulse. On the 23d, the tenesmus continued, and towards the evening an increase of pain of abdomen was relieved by an anodyne enema and warm bath. On the 24th, it is reported that during the night there had been six copious feculent evacuations without tenesmus, and during the day four evacuations, scanty and mucous. On the 25th, five evacuations, feculent and bilious; and at 8, p. m., there was expelled, per anum, a portion of intestine about seven inches in length. Says he was not sensible of its presence in the rectum, but first perceived it when partially expelled, and retained at one end by the sphincter muscle;—the gut appears to be in a putrid state.

The patient continued in the hospital with relaxed and irregular bowels till the 9th of May, when he left much improved in health. He was the carpenter of a ship, and had made twelve voyages to India. Health in general good, and, with the exception of one attack of cholera, had never suffered from serious disease;—there was tendency to constipation, but to no considerable extent; and there never had been suffering from any other affection of the bowels."

Now, as surgeon of the ship, (Prince Regent, Capt. William Bouchier, R.N.) of which the patient alluded to, John Tucker, was carpenter, I beg to say that he was received on board from the hospital on the 9th of May, the day before we sailed from Bombay, certainly a little improved in health, but far from being recovered, having never been able to leave his bed but for one short hour. He lingered on with all the symptoms of chronic dysentery till the night of the 20th July, when he died from extreme weakness and emaciation.

I exceedingly regret that circumstances at that time prevented me from making a post-mortem examination.

You remark there may reasonably be entertained some doubts as to the fact of the above being actually a piece of intestine, since it is reported that the patient recovered. I saw the piece in question, and it appeared to me to be really a portion of intestine, but then he did not recover after voiding it as reported.

I am, Gentlemen, your obedient Servant,

DAVID FIDDES, Surgeon.

---

### Purpura Hæmorrhagica fatal in Five Days.

Mr. De B——, aged 18, came home apparently well on Friday, 21st Dec. and ate his dinner. In the night he was seized with severe headache and rigor; and next morning Mr. Hodding gave him some calomel and a black dose. Next day (Sunday) he had a severe rigor again, with violent pains in the limbs, and that evening the face got red, but without any petechiæ. The redness spread downwards, and petechiæ appeared on the chest, and ultimately over the whole body, in the most intense manner, they being in many places, so confluent as to resemble a black surface. The hæmorrhage commenced the next day (Monday) from bowels, kidneys, throat, nose, and even the eyes, to a most frightful extent. Sulphate of magnesia and well acidulated infusion of roses were given so as to clear the bowels, and acids were freely exhibited with the view of restraining the hæmorrhage, but to no purpose. On Tuesday morning the patient was seen by Dr. M. Hall and Mr. Hodding, and the case appeared to Dr. Hall to be a complication of malignant scarlatina with purpura hæmorrhagica. A grain of sulphate of quinine was ordered every hour with mineral acids. Dr. Johnson saw the patient at three o'clock, and afterwards at ten in the evening of the same day, with Mr. Hodding. The spectacle on this day, which the patient exhibited, was truly awful. The features were so swelled, and the face so covered with black coagulated gore, that no person could have recognized him. The blood was issuing from eyes, nose, mouth, bladder, and bowels. The skin was either black with confluent petechiæ, exhibiting extensive tracts of ecchymosis, or densely covered with dark petechiæ. The pulse was 120, and rather firm, in the morning—the intellect clear—the tongue rather pale, as were the fauces, where not covered with blood; and the fætor from the breath was scarcely bearable. The chest was sonorous throughout—the breathing not laborious—the abdomen soft—and the skin not much above the natural temperature. Some sulphate of magnesia was given this day, with the acid draughts, which cleared the bowels, and in the evening, as the pulse was flagging, the quinine was again resumed, with tincture of hop, beef-tea, &c. The blood still continued to flow from all the mucous membranes in large quantities.

At 10 o'clock on Wednesday morning, the patient was found in a still worse condition than before. The pulse was quick and intermitting—the blood issuing from all the outlets—yet he had slept some, and was able to get out of bed to the night-chair. Port wine was ordered every hour, with powerful mineral acids and quinine. At 4 o'clock the symptoms had rather increased, and at 10 o'clock at night he expired.

The pathology of this disease is involved in Cimmerian darkness. Is the fault in the fluids, or the solids—or both? No one can answer the question. It is not a little mysterious that, in the course of a few hours, both the blood and its containing vessels should be so changed that every capillary vessel, on the external as well as internal surfaces, should pour out the vital fluid, as though it were oozing through a piece of thin muslin! Would venesection have been beneficial in the beginning of this terrible disease? I fear that it would not. A local hæmorrhage, as from the lungs or the stomach, may be mitigated by venesection; but it is a very different thing where the whole capillary system, inside and out, is allowing the blood to escape. In purpura hæmorrhagica, the flow of blood is not caused by mere *vis a tergo*, or the rupture of larger or smaller vessels in a particular locality; whatever be the pathological condition, it is general and not topical. If plethora were the cause, surely the hæmorrhage would stop before all the blood of the body was drained off! There appears to be some analogy between this disease and malignant cholera. In the latter case the serum of the blood exudes through the capillaries of the mucous membrane of the intestines, until only thick tarry blood is left in the larger vessels. In purpura, the blood itself, red and white portions, exudes not only through the intestinal capillaries, but through all capillaries, till the patient dies of the hæmorrhage!

Until we know something of the pathology of the disease, whether in the solids or the fluids, there is little chance of discovering a successful method of treatment.

## Figures and Tables

**Figure f1:**